# The association between high-sensitivity C-reactive protein at admission and progressive motor deficits in patients with penetrating artery infarctions

**DOI:** 10.1186/s12883-019-1538-5

**Published:** 2019-12-29

**Authors:** Pengyu Gong, Yukai Liu, Ting Huang, Wenxiu Chen, Teng Jiang, Yachi Gong, Min Lu, Meng Wang, Yingdong Zhang, Xiaohao Zhang, Qiwen Deng, Junshan Zhou

**Affiliations:** 1 Department of Neurology, Nanjing First Hospital, Nanjing Medical University, No. 68 Changle Road, Nanjing, 210006 Jiangsu China; 2 Department of Critical Care Medicine, Nanjing First Hospital, Nanjing Medical University, No.68 Changle Road, Nanjing, 210006 Jiangsu China; 3 Department of Gerontology, Nantong Third Peoples Hospital, Nantong University, 60 Mid-Youth Road, Nantong, 226006 Jiangsu China; 4 Department of Neurology, Second Affiliated Hospital of Nanjing University of Chinese Medicine, Nanjing, 210002 Jiangsu China

**Keywords:** Progressive motor deficit, Penetrating artery infarction, High-sensitivity C-reactive protein, Ischemic stroke

## Abstract

**Background:**

A fraction of patients with penetrating artery infarction (PAI) experience progressive motor deficit deterioration (PMD). We sought to investigate the role of high-sensitivity C-reactive protein (hs-CRP) at admission in predicting PMD.

**Methods:**

From January 2015 to September 2018, consecutive patients with PAI from three centers were prospectively enrolled in this study. PMD was defined as worsening of motor function score by ≥1 point on the National Institutes of Health Stroke Scale during the first 5 days after admission. Multivariable logistic regression analyses were performed to explore the relationship between hs-CRP and PMD in patients with PAI. We also performed receiver operating characteristic curve analysis and constructed a nomogram to assess the overall discriminative ability of hs-CRP in predicting PMD.

**Results:**

We ultimately included 544 patients (mean age, 65.4 ± 11.8 years). A total of 85 (15.6%) patients were identified to have PMD. Multivariate logistic regression analysis showed that hs-CRP was independently associated with PMD (*P* = 0.001). The optimal cutoff value for hs-CRP as a predictor for PMD was 3.48 mg/L, with a sensitivity of 73.64% and a specificity of 82.35% (area under curve, 0.792). Moreover, the nomogram we constructed indicated that higher level of hs-CRP was an indicator of PMD (c-index = 0.780, *P* < 0.001).

**Conclusions:**

Our study suggested that hs-CRP might be a useful biomarker for predicting the risk of PMD in patients with PAI.

## Introduction

Ischemic stroke is one of the leading causes of mortality and disability worldwide [1–3]. Progressive motor deficit (PMD) is one of the most common neurological deterioration during the acute stage of penetrating artery infarction (PAI), which accounts for almost 25% of all ischemic stroke [4]. The incidence of PMD ranges from 13 to 38% in patients with PAI [5–8]. Several reports have shown that PMD is also associated with poor prognosis of PAI [9–11]. Although several biomarkers [4, 12] have been identified in previous studies, PMD remains insidious and largely unpredictable in clinical practice. Therefore, exploration of the potential mechanisms and measurable biomarkers of PMD among patients with PAI is important.

Neuroinflammatory processes play a fundamental role in the acute stage of ischemic stroke [13–15]. Several inflammatory biomarkers was reported to be correlated with neurological deterioration in patients with acute ischemic stroke, such as lipoprotein-associated phospholipase A2, [16]. neutrophil–lymphocyte ratio [17] and so on. Previous studies have revealed that high-sensitivity C-reactive protein (hs-CRP) may act as an inflammatory factor that responds to ischemic stroke [18, 19]. A high hs-CRP level has been found to show predictive value for poststroke depression [18] and poor outcome [19–22] in ischemic stroke patients. The levels of hs-CRP may be associated with the risk of excessive ischemic stroke independently [23]. However, there are few studies that focused on the clinical value of hs-CRP in patients with PAI. The association between hs-CRP and PMD in ischemic stroke remains unclear. Thus, the purpose of this tricenter observational study was to assess the association between hs-CRP levels at admission and PMD in patients with PAI.

## Methods

### Patient selection

Consecutive patients who presented with symptoms of a lacunar syndrome between January 2015 and September 2018 underwent a standard in-house procedure [24, 25] and prospectively recruited from three hospitals. All the patients were treated in the stroke units and received treatments, such as antiplatelet therapy statin therapy and risk factor management. Magnetic resonance (MR), computed tomography, electrocardiogram, echocardiography, carotid ultrasonography and transcranial Doppler, and were performed for assessing the stroke etiology. Eligible patients were included in the present analysis if they met the following criteria.

The inclusion criteria were as follows:

1. admission within 24 h of onset with a lacunar syndrome;
2. patients with penetrating artery infarctions;
3. age more than 18 years.

The exclusion criteria were as follows:

1. Patients who had a potential source of cardioembolism or >50% stenosis of the extracranial carotid artery;
2. severe inflammatory diseases or infectious diseases;
3. lack of motor deficits, such as patients with pure sensory syndrome;
4. renal failure or hepatic failure;
5. medical history of Parkinson’s disease or other dyskinesia;
6. the neurological deficits of patients cannot be evaluated over the following 5 days after admission.

### Vascular risk factors

Hypertension was defined as systolic blood pressure (SBP) ≥140 mmHg/or diastolic blood pressure (DBP) ≥90 mmHg or use of antihypertensive medication within 2 weeks. SBP and DBP were measured and recorded soon after admission. Diabetes mellitus was defined as either a fasting blood glucose (FBG) level > 7.0 mmol/L on more than two occasions or the use of an antidiabetic medication. Dyslipidemia was defined as a total cholesterol level > 5.70 mmol/L and/or a triglyceride level > 5.18 mmol/L on more than two occasions or the use of lipid-lowering agents. Current smoking and drinking habits were defined as regular smoking and/or drinking at the time of stroke, respectively.

### MR imaging

All participants underwent MR imaging (MRI) and MR angiography (MRA). MRI scans were performed with 3.0-T superconducting magnets. Intracranial artery vessels, including the middle cerebral arteries (MCAs) and vertebrobasilar arteries (VBAs), were assessed by MRA. The severity of stenosis in each intracranial artery was graded based on maximal luminal narrowing according to the following criteria: normal, mild stenosis (< 50%) and moderate or severe stenosis (50% or more).

The severity of carotid artery atheromatosis was graded based on the examination results of carotid ultrasonography, which was divided into the following three categories: absence, moderate (< 70%) and severe (70% or more).

### Definition of penetrating artery infarction and progressive motor deficit

PAI was defined as a relevant deep, single hyperintensity in the territory of penetrating arteries 20 mm or less in diameter on axial slices of an MRI with diffusion-weighted imaging (DWI) that corresponded to one of the lacunar syndromes during a patient’s presentation in the acute phase.

The evaluation of neurological deficits was conducted using the National Institutes of Health Stroke Scale (NIHSS) score on admission and continued over the following 5 days 2–3 times every day after admission by two certified neurologists blind to clinical information.

PMD was defined as worsening of motor function by ≥1-point on the motor section of NIHSS during the first 5 days after admission [12, 26–28].

White matter lesions were defined as diffuse hyperintensities that were located in the subcortical and periventricular white matter on T2-weighted images and proton density images. Silent lacunar infarcts were defined as penetrating artery occlusions 3 to 15 mm in diameter in horizontal sections with high intensity on both T2-weighted images and DWI.

### Masurement of hs-CRP

All the blood samples were collected at 7 AM the second day after admission. The levels of hs-CRP were measured with an immunoturbidimetry assay on an Architect c16000 chemistry analyzer (Abbott Diagnostics, Abbott Park, USA).

### Statistical analysis

Statistical analyses were performed with SPSS version 21.0 (SPSS Inc., Chicago, IL, USA). Continuous variables that followed a normal distribution were expressed as the mean ± standard deviation; other continuous variables that did not follow normal distributions were presented as the median and interquartile range (25th to 75th percentile). Categorical variables were expressed as constituent ratios. Differences in baseline characteristics among the hs-CRP quartiles were tested using analysis of variance or the Kruskal-Wallis test for continuous variables, and Pearson’s chi-square test for categorical variables. We also used binary logistic regression analysis to detect the risk factors for PMD. Multivariable analysis was adjusted for all potential confounders with a statistically significant association at *P* < 0.05 in univariate regression analysis. Receiver operating characteristic (ROC) curve analysis was performed to assess the overall discriminative ability of hs-CRP to predict PMD and to establish optimal cutoff points at which the sum of the specificity and sensitivity was the highest. A MedCalc 15.6.0 (MedCalc Software Acacialaan 22, B-8400 Ostend, Belgium) packet program was used to obtain the ROC curve and to analyze specify and sensitivity of hs-CRP for the exitus status. In addition, a nomogram based on the independent predictors was constructed by R software with the package rms. The predictive capacity of the nomogram was determined by Harrell’s c-index. A two-tailed value of *P* < 0.05 was considered significant.

## Results

**Fig. 1:**
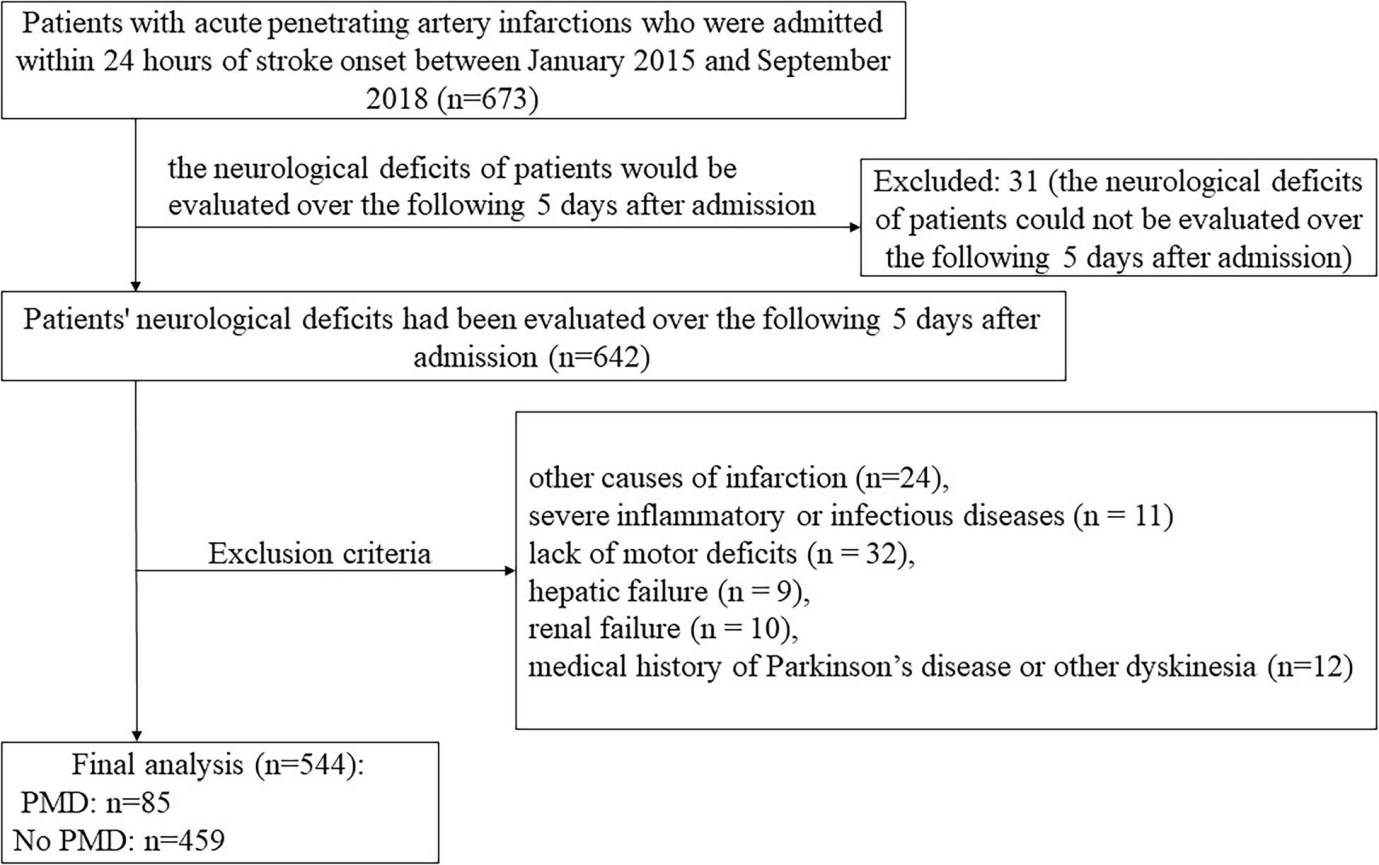
Patient flowchart

From January 2015 to September 2018, 642 patients with acute PAI who were admitted within 24 h of stroke onset were screened for 5 days in this study (Fig. 1). Thirty-one patients’ neurological deficits could not be evaluated over the following 5 days after admission. Sixty-eight patients were excluded for the following reasons: other causes of infarction (*n* = 24), severe inflammatory or infectious diseases (*n* = 11), lack of motor deficits, such as patients with pure sensory syndrome (*n* = 32), hepatic failure (*n* = 9), renal failure (*n* = 10), medical history of Parkinson’s disease or other dyskinesia (*n* = 12). A total of 544 subjects (387 men; mean age, 65.4 ± 11.8 years) were included in the final analysis (Fig. 1). PMD was observed in 85 patients (15.6%).

A comparison of the baseline characteristics of the groups with and without PMD are presented in Table 1. The PMD group had significantly higher levels of hs-CRP than the non-PMD group (5.9 [4.0, 19.8] versus 2.0 [1.3, 3.8], *P* < 0.001).

**Table 1:** Baseline Characteristics of Patients with PMD and Non-PMD

Variable	PMD (*n* = 85)	Non-PMD (*n* = 459)	*P*
Demographic characteristics			
Age, years	67.8 ± 11.6	64.9 ± 11.8	0.038
Male, %	60 (70.6)	327 (71.2)	0.903
Vascular risk factors, %			
Hypertension	56 (65.9)	304 (66.2)	0.950
Diabetes mellitus	44 (51.8)	117 (25.5)	< 0.001
Dyslipidemia	15 (17.6)	76 (16.6)	0.805
Current smoking	30 (35.3)	191 (41.6)	0.276
Current drinking	19 (22.4)	143 (31.2)	0.197
Previous stroke	7 (8.2)	60 (13.1)	0.213
Peripheral artery disease	4 (4.7)	12 (2.6)	0.294
Coronary artery disease	12 (14.1)	54 (11.8)	0.542
Clinical data			
Previous antiplatelet, %	10 (11.8)	47 (10.2)	0.673
Previous statin, %	2 (2.4)	14 (3.1)	0.727
SBP, mmHg	142.8 ± 19.8	144.9 ± 20.8	0.445
DBP, mmHg	84.7 ± 11.7	86.0 ± 12.4	0.449
Body mass index, kg/m2	24.3 ± 4.3	24.5 ± 3.3	0.773
Initial total NIHSS, score	3 (1–4)	2 (2–4)	0.728
White matter lesions, %	60 (70.6)	294 (64.1)	0.246
Intravenous thrombolysis, %	15 (17.6)	76 (16.6)	0.805
Infra-tentorial infarction, %	42 (49.4)	244 (53.2)	0.525
Supra-tentorial infarction, %	43 (50.6)	215 (46.8)	0.525
MCA stenosis, %			
0	46 (54.1)	261 (56.9)	0.639
≤ 50%	35 (41.2)	173 (37.7)	0.544
>50%	4 (4.7)	25 (5.4)	0.780
VBA stenosis, %			
0	40 (47.1)	258 (56.2)	0.577
≤ 50%	31 (36.5)	172 (37.5)	0.861
>50%	9 (10.6)	30 (6.5)	0.183
Carotid artery atheromatosis, %			
Absence	39 (45.9)	183 (39.9)	0.300
Moderate	39 (45.9)	239 (52.7)	0.295
Significant	7 (8.2)	37 (8.1)	0.957
Silent lacunar infarcts, %	42 (49.4)	204 (44.4)	0.398
OMT, day	1 (1,2)	1 (1,2)	0.589
Antibiotic use, %	23 (27.1)	92 (20.0)	0.146
Laboratory data			
Leukocyte number, 10^^9^	8.5 ± 2.4	8.0 ± 4.3	0.348
TC, mmol/L	4.4 ± 1.2	4.5 ± 1.0	0.338
TG, mmol/L	2.0 ± 1.4	1.7 ± 1.1	0.073
HDL, mmol/L	1.0 ± 0.2	1.1 ± 0.4	0.306
LDL, mmol/L	2.7 ± 0.8	2.7 ± 0.8	0.902
FBG, mmol/L	7.5 ± 3.5	5.7 ± 2.1	< 0.001
Glycated hemoglobin, %	7.5 ± 2.4	6.2 ± 1.3	0.002
Homocysteine, umol/L	15.6 ± 8.0	16.1 ± 8.3	0.519
Hs-CRP, mg/L	5.9 (4.0–9.8)	2.0 (1.3–3.8)	< 0.001

Abbreviations: SBP, Systolic blood pressure; DBP, Diastolic blood pressure; National Institutes of Health Stroke Scale; MCA, Middle cerebral artery; VBA, Vertebro-basilar artery; OMT, Onset to the measurement of hs-CRP time; TC, Total cholesterol; TG, Triglyceride; HDL, High density lipoprotein; LDL, Low density lipoprotein; FBG, Fast blood glucose

‘Antibiotic use’ means ‘the antibiotic use during hospitalisation’

The median hs-CRP was 6.46 mg/L, with quartile levels as follows: 0.18 mg/L to 1.28 mg/L (first quartile); 1.28 mg/L to 2.33 mg/L (second quartile); 2.35 mg/L to 5.19 mg/L (third quartile); 5.26 mg/L to 293.00 mg/L (fourth quartile). Baseline characteristics of the study population according to hs-CRP quartiles are provided in Table 2. The results showed that increased hs-CRP was significantly related to PMD in patients with acute PAI (*P* = 0.001).

**Table 2:** Characteristics of subgroups based on the quartile of hs-CRP

Variable	total (*n* = 544)	quartile 1 (*n* = 136)	quartile 2 (n = 136)	quartile 3 (n = 136)	quartile 4 (n = 136)	*P*
Demographic characteristics						
Age, years	65.4 ± 11.8	65.0 ± 10.3	65.8 ± 12.0	63.8 ± 11.6	66.9 ± 13.0	0.169
Male, %	387 (71.1)	94(69.1)	103(75.7)	88(64.7)	102(75.0)	0.145
Vascular risk factors, %						
Hypertension	360 (66.2)	93 (68.4)	90 (66.2)	91 (66.9)	86 (63.2)	0.836
Diabetes mellitus	161 (29.6)	36 (26.5)	36 (26.5)	40 (29.4)	49 (36.0)	0.243
Dyslipidemia	91 (16.7)	25 (18.4)	21 (15.4)	22 (16.2)	23 (16.9)	0.927
Current smoking	221 (40.6)	55 (40.4)	63 (46.3)	47 (34.6)	56 (41.2)	0.270
Current drinking	162(29.8)	47 (34.6)	44 (32.6)	33 (24.3)	38 (27.9)	0.545
Previous stroke	67 (12.3)	18 (13.2)	16 (11.8)	22 (16.2)	11 (8.1)	0.234
Peripheral artery disease	16 (2.9)	3 (2.2)	3 (2.2)	4 (2.9)	6 (4.4)	0.672
Coronary artery disease	66 (12.1)	18 (13.2)	19 (14.0)	15 (11.0)	14 (10.3)	0.760
Clinical data						
Previous antiplatelet, %	57 (10.5)	13 (9.6)	14 (10.3)	17 (12.5)	13 (9.6)	0.839
Previous statin, %	16 (2.9)	5 (3.7)	4 (2.9)	5 (3.7)	2 (1.5)	0.627
SBP, mmHg	144.6 ± 20.6	145.0 ± 19.0	143.1 ± 21.3	143.6 ± 20.5	146.8 ± 21.7	0.465
DBP, mmHg	85.8 ± 12.3	85.5 ± 12.3	84.5 ± 12.5	86.6 ± 11.8	86.5 ± 12.7	0.478
Body mass index, kg/m2	24.5 ± 3.5	24.1 ± 3.7	24.1 ± 3.0	24.9 ± 3.2	24.8 ± 3.9	0.145
Initial totaol NIHSS, score	2 (2–4)	2 (1–4)	2 (2–4)	2 (1–4)	3 (2–4)	0.631
White matter lesions, %	354 (65.1)	94 (69.1)	85 (62.5)	88 (64.7)	87 (64.0)	0.692
Intravenous thrombolysis, %	91 (16.7)	19 (14.0)	22 (16.2)	24 (17.6)	26 (19.1)	0.703
PMD, %	85 (15.6)	7 (5.1)	3 (2.2)	30 (22.1)	45 (33.1)	< 0.001
MCA stenosis, %						
0	307 (56.4)	83 (61.0)	74 (54.4)	73 (53.7)	77 (56.6)	0.611
≤ 50%	208 (38.2)	48 (35.3)	54 (39.7)	54 (39.7)	52 (38.2)	0.862
>50%	29 (5.3)	5 (3.7)	8 (5.9)	9 (6.6)	7 (5.1)	0.735
VBA stenosis, %						
0	302 (55.5)	80 (58.8)	71 (52.2)	73 (53.7)	78 (57.4)	0.664
≤ 50%	203 (37.3)	48 (35.3)	56 (41.2)	51 (37.5)	48 (35.3)	0.719
>50%	39 (7.2)	8 (5.9)	9 (6.6)	12 (8.8)	10 (7.4)	0.809
Carotid artery atheromatosis, %						
Absence	222 (40.8)	46 (33.8)	59 (43.4)	62 (45.6)	55 (40.4)	0.220
Moderate	278 (51.1)	76 (55.9)	69 (50.7)	62 (45.6)	71 (52.2)	0.369
Significant	44 (8.1)	14 (10.3)	8 (5.9)	12 (8.8)	10 (7.4)	0.577
Silent lacunar infarcts, %	246 (45.2)	63 (46.3)	56 (41.2)	62 (45.6)	65 (47.8)	0.721
OMT, day	2 (1,2)	2 (1,2)	2 (1,3)	2 (1,2)	2 (1,2)	0.417
Antibiotic use, %	115 (21.1)	23 (16.9)	32 (23.5)	24 (17.6)	36 (26.5)	0.155
Laboratory data						
Leukocyte number, 10^^9^	8.4 ± 4.1	8.2 ± 2.7	8.2 ± 3.0	8.4 ± 2.6	8.8 ± 6.6	0.562
TC, mmol/L	4.5 ± 1.0	4.3 ± 1.0	4.5 ± 1.1	4.5 ± 1.0	4.4 ± 1.0	0.313
TG, mmol/L	1.8 ± 1.2	1.5 ± 0.8	1.8 ± 1.3	1.9 ± 1.4	1.8 ± 1.1	0.065
HDL, mmol/L	1.1 ± 0.3	1.1 ± 0.5	1.1 ± 0.2	1.0 ± 0.2	1.1 ± 0.3	0.103
LDL, mmol/L	2.7 ± 0.8	2.8 ± 0.8	2.8 ± 0.9	2.8 ± 0.8	2.7 ± 0.9	0.504
FBG, mmol/L	6.0 ± 2.5	5.8 ± 2.2	5.7 ± 2.3	6.3 ± 2.7	6.2 ± 2.6	0.184
Glycated hemoglobin, %	6.4 ± 1.6	6.3 ± 1.4	6.3 ± 1.4	6.6 ± 1.7	6.6 ± 1.7	0.082
Homocysteine, umol/L	16.0 ± 8.2	14.6 ± 6.0	16.9 ± 10.0	15.8 ± 7.5	16.9 ± 8.7	0.076

Abbreviations: SBP, Systolic blood pressure; DBP, Diastolic blood pressure; National Institutes of Health Stroke Scale; MCA, Middle cerebral artery; VBA, Vertebro-basilar artery; OMT, Onset to the measurement of hs-CRP time;TC, Total cholesterol; TG, Triglyceride; HDL, High density lipoprotein; LDL, Low density lipoprotein; FBG, Fast blood glucose

‘Antibiotic use’ means ‘the antibiotic use during hospitalisation’

Table 3 shows the results of logistic regression analysis for risk factors of PMD. Univariable logistic regression analysis was used to investigate the significance of variables on predicting PMD in patients with PAI. Univariate logistic regression analyses demonstrated that the third quartile of hs-CRP, the fourth quartile of hs-CRP, age, diabetes mellitus, and levels of FBG and glycated hemoglobin were associated with PMD (*P* < 0.05). Significant predictors in the univariable analysis were included in a multivariable regression model to determine independent predictors. After adjusting for all potential confounders, age, glycated hemoglobin level and the third quartile and fourth quartile of hs-CRP (first quartile used as the reference value) were identified as independent predictors for PMD.

**Table 3:** Logistic regression analysis for risk factors with PMD

Variable	Unadjusted OR (95%CI)	*P*	Adjusted OR (95%CI)	*P*
Demographic characteristics				
Age, years	1.021 (1.001–1.042)	0.038	1.025 (1.001–1.048)	0.048
Male	0.969 (0.583–1.611)	0.903		
Vascular risk factors				
Hypertension	0.985 (0.604–1.604)	0.950		
Diabetes mellitus	3.137 (1.952–5.041)	0.001	1.757 (0.934–3.304)	0.080
Dyslipidemia	1.080 (0.587–1.987)	0.805		
Current smoking	0.765 (0.473–1.239)	0.227		
Current drinking	0.707 (0.420–1.191)	0.192		
Previous stroke	0.597 (0.263–1.354)	0.217		
Clinical data				
Previous antiplatelet	1.169 (0.566–2.415)	0.674		
Previous statin	0.766 (0.171–3.433)	0.728		
SBP	0.995 (0.983–1.006)	0.374		
DBP	0.992 (0.973–1.011)	0.398		
Body mass index	0.985 (0.913–1.063)	0.694		
Initial total NIHSS	1.018 (0.941–1.103)	0.653		
White matter lesions	1.347 (0.814–2.230)	0.247		
Intravenous thrombolysis	1.080 (0.587–1.987)	0.805		
Infra-tentorial infarction	1.162 (0.731–1.846)	0.525		
Silent lacunar infarcts	1.221 (0.768–1.941)	0.398		
MCA stenosis				
0	1.118 (0.702–1.779)	0.639		
≤ 50%	1.157 (0.722–1.854)	0.544		
>50%	0.926 (0.539–1.590)	0.780		
VBA stenosis				
0	1.141 (0.717–1.815)	0.578		
≤ 50%	0.958 (0.593–1.549)	0.861		
>50%	1.301 (0.879–1.926)	0.188		
Antibiotic use	1.480 (0.871–2.515)	0.147		
Hs-CRP distribution				
Quartile 1	Reference		Reference	
Quartile 2	0.416 (0.105–1.642)	0.211	0.468 (0.110–1.981)	0.302
Quartile 3	5.216 (2.203–12.349)	0.001	5.191(1.974–13.649)	0.002
Quartile 4	9.113 (3.933–21.116)	0.001	9.786 (3.819–25.075)	0.001
Laboratory data				
Leukocyte number, 10^^9^	0.957 (0.879–1.043)	0.320		
TC, mmol/L	0.930 (0.743–1.164)	0.528		
TG, mmol/L	1.169 (0.983–1.391)	0.077		
HDL, mmol/	0.548 (0.220–1.365)	0.196		
LDL, mmol/L	0.876 (0.657–1.169)	0.370		
FBG, mmol/L	1.254 (1.152–1.364)	0.001	1.067 (0.934–1.218)	0.340
Glycated hemoglobin, %	1.495 (1.313–1.701)	0.001	1.351 (1.095–1.668)	0.005
Homocysteine, umol/L	0.992 (0.960–1.026)	0.646		

Abbreviations: SBP, Systolic blood pressure; DBP, Diastolic blood pressure; National Institutes of Health Stroke Scale; MCA, Middle cerebral artery; VBA, Vertebro-basilar artery;TC, Total cholesterol; TG, Triglyceride; HDL, High density lipoprotein; LDL, Low density lipoprotein; FBG, Fast blood glucose

**Fig. 2:**
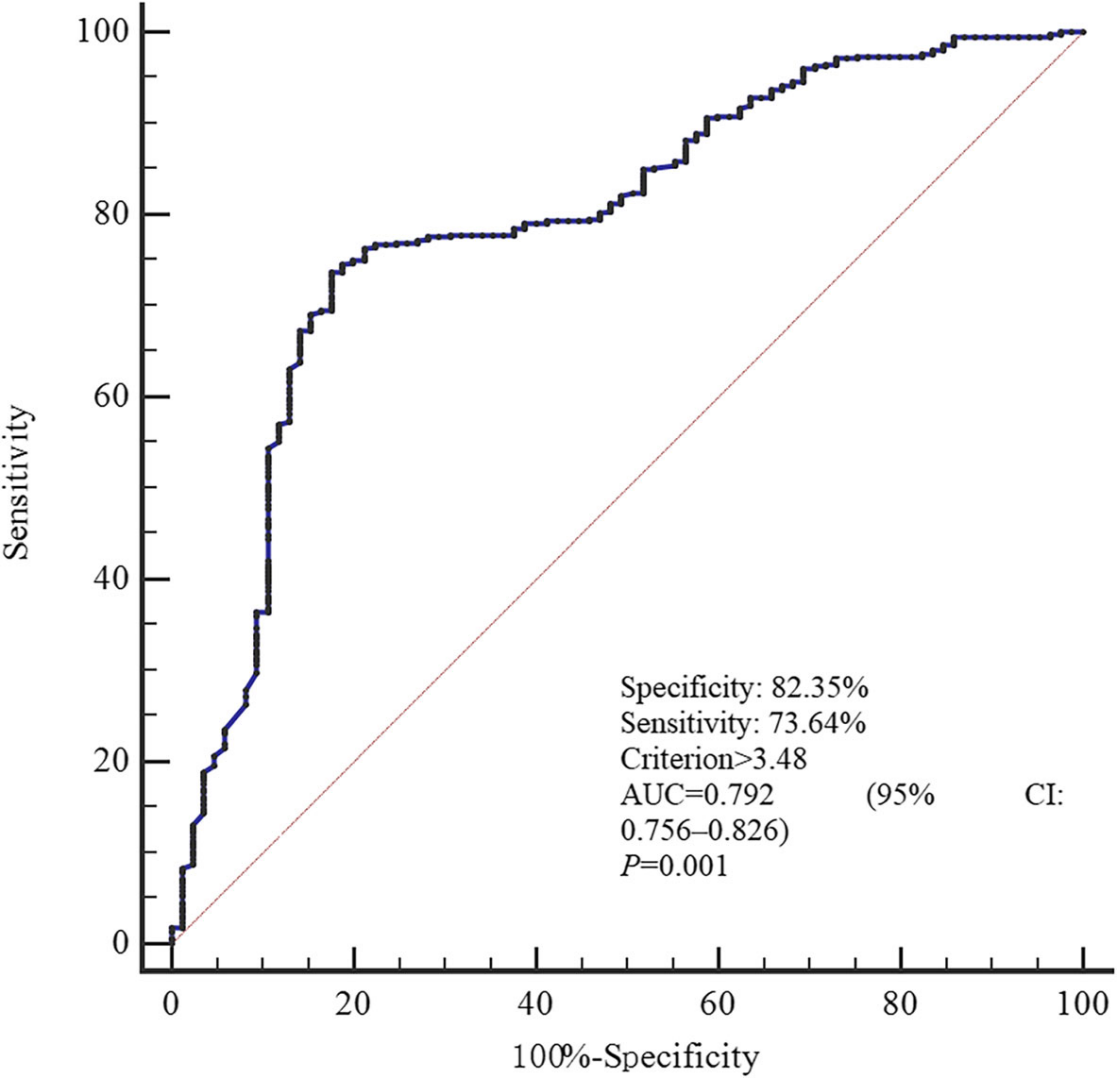
Receiver operating characteristic (ROC) curve for the value of hs-CRP to predict PMD

To further assess the clinical significance of hs-CRP in PMD, we performed a ROC curve analysis as depicted in Fig. 2. We observed that the area under curve (AUC) of hs-CRP was 0.792 (95% CI, 0.756–0.826) with the ability to discriminate PMD. The optimal cutoff value for hs-CRP as a predictor of PMD was determined to be 3.48 mg/L in the ROC curve analysis, yielding the largest Youden’s index value (a sensitivity of 73.64% and a specificity of 82.35%). The AUC was 0.792 (95% CI, 0.756–0.826).

**Fig. 3:**
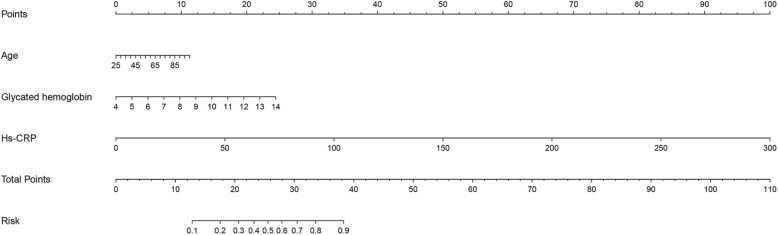
Nomograms of patients with PAI for predicting PMD. Each factor was given a point on the basis of the nomograms. The final total points were obtained by adding the individual score of each of the 3 risk factors. The estimated probability of PMD of the individual patient with PAI can be easily obtained from the nomogram based on the total points

The nomogram is shown in Fig. 3, and the concordance index of this model was 0.780 (*P* < 0.001). These findings were similar to those obtained previously in the multivariate logistic models.

## Discussion

Our observational study revealed that elevated plasma levels of hs-CRP remained an independent predictor for PMD in patients with PAI after adjusting for age, diabetes mellitus and other possible confounders. In general, a biomarker with 0.7 < area under the curve < 0.9 indicates a moderate diagnostic value. High hs-CRP levels (> 3.48 mg/L) have a moderate ability to diagnose PMD. Furthermore, our constructed nomogram indicated that higher hs-CRP was an indicator of PMD. Thus, the hs-CRP value at admission represented a readily available predictor for PMD in patients with PAI. The serum biomarker, hs-CRP at admission, is able to identify earlier than the standard clinical and imaging assessment. Furthermore, our study also showed that age and glycated hemoglobin were predictors of PMD, which was consistent with the findings of other studies [19, 28].

The influence of hs-CRP on ischemic stroke has been well established, and hs-CRP has been reported as a predictor of disease severity, prognosis and mortality in patients with ischemic stroke [19, 29]. Furthermore, high plasma hs-CRP levels are associated with clinical complications following acute ischemic stroke [18, 30] PMD, which may result in severe morbidity, commonly occurs in patients with PAI during the acute stage. This is the first study to explore the relationship between hs-CRP and PMD in patients with penetrating artery ischemic stroke. PMD was revealed to have an incidence of 15.6% in this trial, which was in accordance with a previous study [19, 28]. Moreover, in a previous case presentation, the patient with PMD was found to be complicated by depressive disorder and anxiety disorder [31]. Our observational study showed the predictive value of hs-CRP for the occurrence of PMD in patients with PAI. It provides a biomarker for early detection of PMD.

Hs-CRP, a systemic inflammatory marker, is produced in large amounts by hepatocytes in response to IL-1, IL-6 and TNF-α [32, 33]. Inflammatory responses play a vital role in ischemic stroke [13–15, 34, 35]. The ischemic tissues release inflammatory cytokines and chemokines, among which hs-CRP is one of the mediators of ischemic brain injury. Cytokines and inflammatory factors lead to neuronal necrosis, endothelial permeability of vessels and blood-brain barrier disruption, resulting in the mortality of neurons and induction of apoptosis [34, 35]. Hence, PMD is believed to result from biochemical abnormalities such as inflammation.

However, several limitations should be considered. First, the sample size of our study was relatively small, and larger cohorts of subjects are needed. Second, we did not investigate dynamic changes in hs-CRP; the combination of baseline and dynamic hs-CRP may provide a more objective and comprehensive way to predict PMD in PAI patients. Third, we only performed digital subtraction angiography (DSA) for a limited number of patients. The severity of stenosis in each intracranial artery could only be assessed by by MRA instead of DSA,which may not be the most precise. Moreover, we did not perform the plaque imaging to evaluate carotid artery atheromatosis, which might be a factor that is related to inflammation process. Finally, many factors that might affect inflammatory markers were not taken into consideration.

## Conclusion

In summary, based on the conclusion of our study, hs-CRP levels are able to serve as a useful noninvasive biomarker for the assessment of PMD. The association between hs-CR and PMD should be considered in the management of PAI.

## Data Availability

The datasets used and/or analysed during the current study are available from the corresponding author on reasonable request.

## Abbreviations

AUC: Area under curve
DWI: Diffusion-weighted imaging
FBG: Fasting blood glucose
hs-CRP: High-sensitivity C-reactive protein
MCA: Middle cerebral artery
MR: Magnetic resonance
MRA: Magnetic resonance angiography
MRI: Magnetic resonance imaging
NIHSS: National Institutes of Health Stroke Scale
PAI: Penetrating artery infarction
PMD: Progressive motor deficit
ROC: Receiver operating characteristic
VBA: Vertebrobasilar arteries
